# Discordant Restoration of TCR Expression and Function by CD247 Somatic Reversions

**DOI:** 10.1007/s10875-025-01908-9

**Published:** 2025-07-25

**Authors:** Alejandro C. Briones, Ana V. Marin, Rebeca Chaparro-García, Marta López-Nevado, David Abia, Ivan Estevez-Benito, Daniel Chacón-Arguedas, Edgar Fernández-Malavé, Paula P. Cardenas, José R. Regueiro

**Affiliations:** 1 Department of Immunology, Ophthalmology and ENT, Complutense University School of Medicine and 12 de Octubre Health Research Institute (imas12), 28040 Madrid, Spain; 2 Bioinformatics Unit, Centro de Biología Molecular Severo Ochoa (CSIC-UAM), Campus of the Universidad Autónoma de Madrid, Madrid, Spain

**Keywords:** CD247, CD3Z, TCR, Immunodeficiency, Somatic reversions

## Abstract

**Background:**

The CD247 chain of the T-cell receptor (TCR) is essential for normal T cell development and function. Reported CD247-deficient patients showed severe immunodeficiency despite the presence of two populations of peripheral T cells, most with low TCR levels carrying the germline variant and a few with higher TCR levels due to somatic reversion. However, the revertant T cells remained a minority and did not improve the patients’ clinical status.

**Purpose:**

To compare the capability of somatic revertant variants of CD247 germline changes (p.M1T and p.Q70X) to restore TCR expression and function.

**Methods:**

CD247 wild-type (WT) and p.Q70L/W/Y somatic variants were individually introduced in CD247-deficient mouse (MA5.8), human mutant (PM1T), and CRISPR/Cas9-generated Jurkat (ZKO) T cell lines by nucleofection or transduction.

**Results:**

MA5.8 mouse T cells do not accurately model human CD247 deficiencies, as Q70X restores TCR expression in MA5.8 but not in human cells. In human cell models, all somatic revertant variants restored TCR expression with varying degrees (WT = Q70L > Q70W > Q70Y). In contrast, TCR-induced activation events, such as CD69/CD25 upregulation, showed a different hierarchy (WT = Q70W > Q70L = Q70Y). Furthermore, all CD247 somatic variants failed to induce TCR-mediated ZAP70 tyrosine phosphorylation compared to WT.

**Conclusion:**

Somatic reversions, such as those detected in patients with pathogenic CD247 germinal changes, display a discordant capability to rescue TCR expression versus function. These findings shed light on the role of CD247 in TCR expression and function during human T cell development, with implications for immunodeficiencies, as well as for the biological consequences of CD247 somatic mosaicism.

**Supplementary Information:**

The online version contains supplementary material available at 10.1007/s10875-025-01908-9.

## Introduction

The CD247 chain, also known as CD3ζ, is crucial for T cell development and function [1]. It plays a key role in T-cell receptor (TCR) assembly and surface expression by cooperating with other invariant CD3 molecules and in TCR development and function by transducing signals from the TCR to initiate the activation and response of T cells for thymus selection or antigen recognition [2, 3].

CD247 deficiency is a rare early-onset primary immunodeficiency disorder characterized by variants in the *CD247* gene (Table S1). These variants can result in a lack of functional CD247 chain expression in T cells causing severe defects of TCR expression and function, which, in turn, may affect T cell development and function and, thus, immune responses. All reported patients showed T cell lymphopenia, suffered infections, and required stem cell transplantation to survive. Older patients also showed autoimmune features, suggesting poor T-cell selection.

Revertant somatic mosaicism is a phenomenon where spontaneous genetic corrections or compensations occur in a subset of cells, partially restoring the function of an altered germ-line gene [4]. CD247 deficiency frequently associates with revertant somatic mosaicism (4 of 5 patients, Table S1), likely due to the propensity of CD247 to vary [5]. This can lead to improvements in TCR expression (partial phenotypic reversion) in a minute subset of T cells, but it is unclear if phenotypic reversion leads to functional reversion, as CD247 protein domains involved in the former may not be sufficient for the latter. The clinical impact of CD247 somatic mosaicism also remains unclear, as it did not improve the clinical status of any reported patient.

T-cell models are invaluable tools for studying human T-cell immunodeficiencies [6]. However, a human T cell model to study CD247 somatic mosaicism has not been reported. MA5.8, a murine CD247-deficient T cell line [7], has been widely used to establish the role of CD247 in TCR assembly [8, 9] and expression [5], as well as to study CD247 splicing variants [10]. As human and murine invariant TCR-associated molecules show differential roles in each species [11, 12], murine T cell models may show limitations to predict human CD247 role in TCR expression and function.

Here, we compared the capacity of CD247 somatic variants in two immunodeficiency cases with severe CD247 germline changes (p.M1T and p.Q70X, Fig. S1) to restore TCR expression and function in different T cell models, both human and murine. The somatic variants in each case were a wild-type (WT) reversion of p.M1T and three missense compensating variants (p.Q70L, p.Q70W, and p.Q70Y) of the germinal p.Q70X.

## Materials and Methods

### Cell Lines and Culture

HTLV-1 transformed p.M1T T-cell line was derived from peripheral blood mononuclear cells (PBMC) of a CD247-deficient patient [13] as described in the Online Repository. The Jurkat wild-type T-cell line (J77cl20 clone) was provided by Dr B. Rubin (Centre National de la Recherche Scientifique, Centre Hospitalier Universitaire, Purpan, Toulouse, France). The Jurkat CD247-deficient cell line, ZKO, was generated by CRISPR/Cas9 gene editing [14]. All human T-cell lines were grown in RPMI-1640 (Lonza, Basel, Switzerland), supplemented with 10% FBS, 1 × L-Glutamine 200 mM, and 1 × Antibiotic Antimycotic from Gibco (Bethesda, MD, USA). In addition, 100 U/mL recombinant human IL-2 (provided by Craig W. Reynolds, Frederick Cancer Research and Development Center, National Cancer Institute, National Institutes of Health, Frederick, MD, USA) was added to p.M1T cells.

The HEK293T and Phoenix-Ampho (ATCC® CRL-3213™) packaging cell lines were grown under standard conditions in Iscove’s Modified Dulbecco’s Medium (Lonza).

The murine T-cell lines, including parental 2B4 and its CD247-deficient derivative MA5.8 [7], were gifted by Balbino Alarcón (Centro de Biología Molecular Severo Ochoa, Consejo Superior de Investigaciones Científicas, Universidad Autónoma de Madrid, Madrid, Spain). These cell lines were cultured in the same conditions as above but with 5% FBS. All cell lines were maintained at 37 °C and 5% CO_2_ in a humidified incubator.

### Plasmids, Nucleofection, Retroviral and Lentiviral Transduction

The pEGFP-N1 vector (Clontech, Mountain View, CA, USA) containing the human CD247 transcript variant or isoform 2 sequence (NM_000734; hCD247-WT from now on) or germline p.Q70X change (X for short) was a kind gift from Hugh T. Reyburn (National Centre for Biotechnology, Madrid, Spain). The somatic variants p.Q70L, p.Q70W and p.Q70Y were introduced by site-directed mutagenesis. For nucleofection assays, 1.5 × 10^6^ immortalized HTLV-1 or murine T cells were nucleofected with 2 μg of pEGFP-N1 plasmid, carrying each CD247 variant, using the Cell Line Nucleofector Kits V or R, respectively, and the Amaxa Nucleofector 2b device (Lonza, Walkersville, MD, USA) according to the manufacturer’s instructions. Twenty hours post-nucleofection, cells were collected, and the transfection efficiency was analyzed through flow cytometry by counting the fraction of green fluorescent protein (GFP)—expressing cells.

For retroviral transduction hCD247-WT, along with germline and somatic CD247 variants were introduced into the pLZRS-IRES-ΔNGFR retroviral plasmid and transfected into Phoenix-AMPHO cells as described [15]. Both the pLZRS-IRES-ΔNGFR retroviral plasmid and the Phoenix-AMPHO cells were provided by Rubén Martínez-Barricarte (Vanderbilt Institute for Infection, Vanderbilt University Medical Center, Nashville, TN).

For lentiviral transduction, the studied CD247 variants were cloned into the pHRSIN-C56W-UbEM lentiviral plasmid (gifted by Hugh T Reyburn). Viral particle generation and cell transduction were performed following the previously reported protocol [14]. Transduced cells were selected by flow cytometry. Those transduced with the pLZRS-IRES-ΔNGFR plasmid were positive for the CD271 marker, whereas those transduced with the pHRSIN-C56W-UbEM plasmid were positive for GFP.

### Flow Cytometry Analysis

Standard extracellular flow cytometry was performed with monoclonal antibodies (mAbs) against human CD3ε (clone UCHT1) from Beckman Coulter (Brea, CA) or mouse CD3ε (clone 145-2C11) from eBioscience (San Diego, CA). In addition, a mAb against CD271 (clone C40-1457) from BD Biosciences (San Jose, CA) was also employed. Intracellular stainings were done with the FOXP3/Transcription factor staining buffer set from Invitrogen. For intracellular quantification, human CD247 mAb (Clone 6B10.2) from BioLegend (San Diego, CA) was used.

To analyze surface TCR complex expression, mAb against TCRαꞵ (clone IP26) from Thermo Fisher Scientific, and TCR Vꞵ8 (clone 56C5.2) from Beckman Coulter, were used.

Data were acquired with a FACSCalibur flow cytometer (BD Biosciences) and analyzed with FlowJo software from TreeStar (Ashland, OR). Cell sorting was carried out to purify specific cell populations using a FACSAria™ III sorter (BD Life Sciences, San Jose, CA). In all cases, mean fluorescence intensity (MFI) stands for geoMFI.

### Functional Studies

CD247 plays a critical role in T cell activation at the initiation of TCR signaling [16]. Proliferation, in contrast, is a late consequence of T cell activation and occurs distally from CD247. Also, the proliferation of transformed T cells (as the ones used in our study) following TCR stimulation does not differ significantly from that of unstimulated cells because of the intrinsic proliferative phenotype, which is usually associated with cell transformation. For these reasons, we chose to assess immediate (ZAP-70 tyrosine phosphorylation) and early (CD69/CD25 surface upregulation) events of TCR-mediated T cell activation to dissect the differential impact of CD247 variants.

To measure CD69 upregulation after TCR engagement, CD247-deficient human T cell lines (HTLV-1 or Jurkat-derived) were stimulated for 24 h with 1 μg/ml of plastic-coated anti-CD3ε (clone OKT3) from eBioscience or 10 ng/ml Phorbol 12-myristate 13-acetate (PMA) plus 1 µM Ionomycin from Sigma-Aldrich.

To analyze T-cell function in a more physiological way (namely, superantigen recognition), WT or ZKO-transduced Jurkat T cells expressing different CD247 variants were co-cultured with Raji cells (Ratio 1:1) preloaded with Staphylococcal Enterotoxin E (SEE) (0,5 μg/mL) (Sigma-Aldrich) for 18 h in round-bottom 96-well plates. Cells were then collected and stained with anti-CD19 (clone HIB19; BD Biosciences) to discriminate Jurkat from Raji cells. CD69 and CD25 induction in response to TCR activation were evaluated essentially as published [14].

ZAP-70 phosphorylation was determined by intracellular flow cytometry. 0.3 × 10^6^ WT or ZKO-transduced Jurkat T cells were stimulated with 20 μg/mL of anti-CD3ε mAb (clone OKT3) for 30 min at 4ºC. Then, anti-CD3ε was crosslinked with 10 μg/mL goat F(ab’)_2_ anti-mouse Ig (H + L) for 5 min at 37 ºC. The reaction was stopped by adding cold PBS and centrifuging at 10.000 rpm for 5 s. Cells were then fixed/permeabilized with eBioscience™ Foxp3/Transcription Factor Fixation/Permeabilization Concentrate and Diluent (Invitrogen), according to the manufacturer’s instructions, and finally stained with anti-ZAP70/Syk (Tyr319, Tyr352) mAb (clone n3kobu5) APC-labelled from eBioscience.

### Statistical Analysis

To determine the statistical significance of the obtained results, either one-sample t-test or one-way ANOVA test was performed. The test used in each case is indicated in the figure legend. The error bars represent the standard error of the mean (SEM).

## Results

### Partial Surface TCR Reconstitution After Transfection of the PM1T Cell Line with hCD247-WT

After confirming that the PM1T cell line lacks CD247 protein, as evidenced by its impairment in TCR expression and function (Fig. S2), it was selected as the cellular model to study the impact of different CD247 variants on TCR assembly and signaling.

First, human CD247-WT (hCD247-WT) was cloned into the pEGFP-N1 vector and then nucleofected into the CD247-deficient human (PM1T) or mouse (MA5.8) cell lines.

**Fig. 1:**
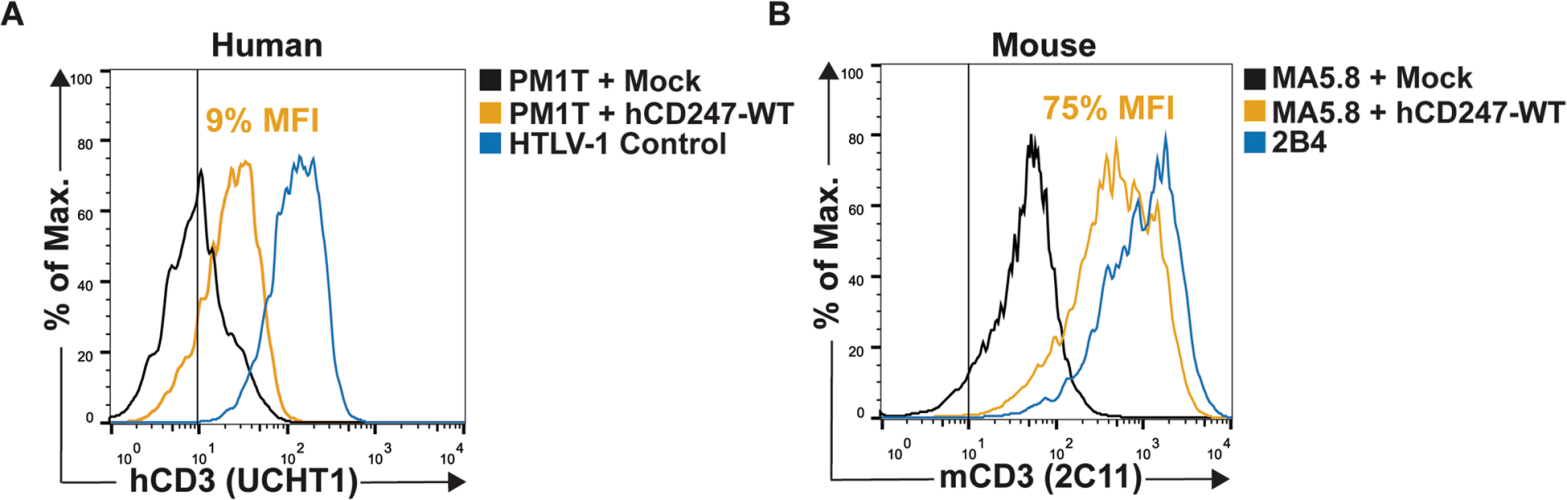
Recovery of surface TCR expression after introducing hCD247-WT into PM1T or MA5.8 cell lines. (**A**) The human CD247-deficient PM1T cell line was transfected by nucleofection with either an empty (Mock) or hCD247-WT carrying construction. A CD247-sufficient HTLV-1 immortalized cell line was used as control of TCR expression. (**B**) Murine CD247-deficient MA5.8 cell line was transfected by nucleofection with either an empty (Mock) or hCD247-WT construction. The CD247-sufficient 2B4 parental cell line was used as a control for TCR expression. In (**A**) and (**B**) surface TCR levels were measured on pre-gated GFP + cells by flow cytometry using mAbs against CD3ε (clones UCHT1 or 2C11 for human or mouse cell lines, respectively). The vertical line represents the isotype control. The graphics represent one experiment (from 2 independent experiments)

Our results showed that hCD247-WT poorly restored surface TCR expression in PM1T, only increasing it from 4 to 9% (relative to HTLV-1 control cell line levels) (Fig. 1A). However, introducing hCD247-WT in MA5.8 significantly improved surface TCR expression, which increased from 12 to 75% (compared to the 2B4 parental cell line) (Fig. 1B).

### Human, but not Mouse, CD247-Deficient Cell Lines Recapitulate CD24 7 Variants’ Effects *in vivo*

**Fig. 2:**
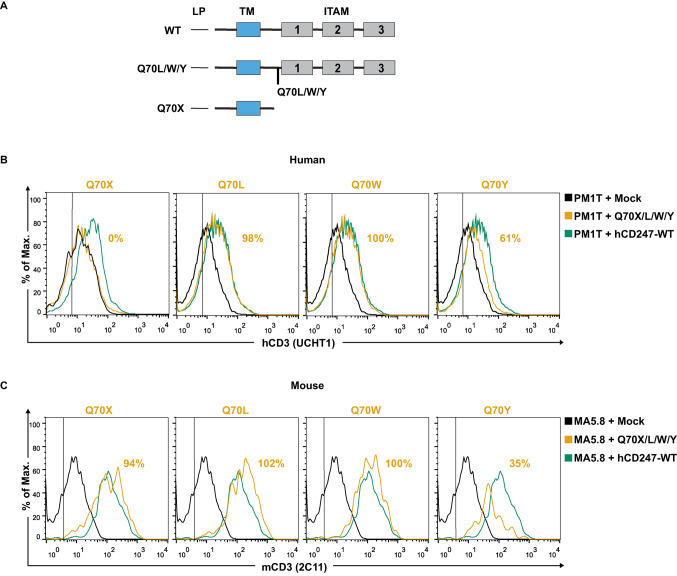
TCR reconstitution ability of the studied hCD247 variants in CD247-deficient human or mouse cell lines. (**A**) Schematic representation of the domain organization of hCD247-WT, somatic reversions Q70L, Q70W and Q70Y, as well as germline change Q70X. The leader peptide (LP), transmembrane region (TM), and immunoreceptor tyrosine-based activation motif (ITAM) are depicted. (**B**) Recovery of TCR surface expression after transfection with the studied hCD247 variants in transfected (GFP +) PM1T or (**C**) MA5.8 cell lines. In (**B**) and (**C**), surface CD3 quantified as MFI% relative to hCD247-WT expression was analyzed with mAbs against hCD3ε (clone UCHT1) or mCD3ε (clone 2C11). The vertical line corresponds to the isotype control. The graphics correspond to one representative experiment (from 3 independent experiments)

For the study of the impact of CD247 variants on surface TCR expression and function, we selected the variants reported in a 10-month-old child with severe combined Immunodeficiency (SCID) caused by CD247 deficiency [17]. These include a CD247 homozygous nonsense germline change, Q70X (which generates a protein lacking all three ITAMs), and was identified in 90% of patients’ T cells that exhibited low TCR expression. Additionally, the study identified three somatic missense reversions, namely, Q70W, Q70L and Q70Y (Fig. 2A), in the remaining 10% of the child’s T cells. These mixed reversions, purified by their higher surface CD3 expression, allowed normal TCR expression but were jointly considered poorly functional by the authors, based on the lack of ZAP-70 phosphorylation upon CD3 engagement [17]. Interestingly, the *in silico* predictors PolyPhen, SIFT or Saphetor classified the Q70X variant as strongly pathogenic, and Q70W and Q70Y variants as probably damaging, whereas Q70L was considered benign.

Nucleofection results showed that the Q70X change did not restore TCR expression in human PM1T, mimicking the patient’s conditions where it was described. Surprisingly, this change fully restored TCR levels in MA5.8 (Fig. 2B, C). Therefore, this result suggests that PM1T is a more suitable model for studying human CD247 variants than the mouse MA5.8 cell line.

Nucleofection assays with plasmids encoding for somatic reversions, Q70W and Q70L, rescued almost up to 100% surface TCR levels in p.M1T (relative to those obtained with the WT gene). However, the Q70Y variant was not able to reach the same level of restoration (61%, compared to those obtained with the WT gene) (Fig. 2B). Similar results were obtained in MA5.8 with Q70W and Q70L variants (Fig. 2C). However, in MA5.8, Q70Y showed a more significant defect in recovering surface TCR than in PM1T (35% vs. 61%, respectively).

Finally, we also found that the nucleofection of Q70X in control Jurkat T cells results in a decrease in surface TCR expression. However, this effect was not observed in control mouse T cells (2B4) (Fig. S3).

### Transduction of CD247-Defective Cell Lines Significantly Improves TCR Surface Reconstitution Values

**Fig. 3:**
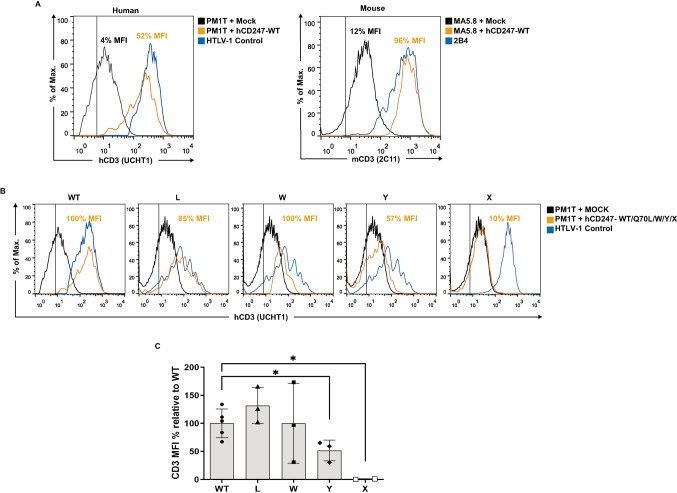
Retroviral transduction of CD247-deficient human (PM1T) or mouse (MA5.8) cell lines. (**A**) Surface TCR complex expression on PM1T or MA5.8 transduced cells (CD271+) was analyzed with mAb against human (UCHT1) or mouse (2C11) CD3, respectively. The vertical line represents the isotype control. (**B**) Surface TCR expression of PM1T cell line transduced with a retroviral plasmid carrying hCD247-WT, Q70L/W/Y somatic reversions and Q70X change. In (**A**) and (**B**), the graphics are representative of a single experiment (from 5 independent experiments). (**C**) Quantification of hCD3 expression from (**B**). MFI values were normalized as: (test-mock)/(WT-mock) (*n* = 3). *p-value < 0.05. Statistical significance was calculated using a one-sample t-test analysis comparing the different variants with respect to hCD247-WT

To improve the TCR reconstitution levels obtained by nucleofection, we cloned the analyzed CD247 variants into the retroviral plasmid pLZRS-IRES-ΔNGFR. Our results showed that transduction with hCD247-WT resulted in a significant increase in TCR expression in PM1T, from 4 to 52%. Meanwhile, the TCR recovery in MA5.8 was almost complete, reaching up to 96%, which is similar to the parental 2B4 cell line levels (Fig. 3A).

When the ability of transduced CD247 somatic reversions to recover TCR expression in PM1T was tested, Q70W rendered similar reconstitution levels to hCD247-WT (100%), whilst Q70L and Q70Y recovered 85% and 57%, respectively. In contrast, Q70X only re-established 9,8% of surface TCR expression (Fig. 3B). The analysis of CD3 MFI values showed that reconstitution levels observed among hCD247-WT and Q70Y/Q70X were statistically significant (Fig. 3C).

According to our data, we have confirmed that transduction is a more efficient method than nucleofection for restoring surface TCR levels in CD247-deficient cell lines. Unfortunately, the retroviral transduction efficiency was very low (8%), which greatly hindered our ability to conduct functional assays with the variants of interest.

As one of the objectives of this work was to test the functional performance of the different CD247 variants, we generated a CD247-deficient Jurkat cell line (ZKO) by employing CRISPR/Cas9 technology. As reported, the ZKO cell line lacked intracellular CD247 and showed a significant reduction in extracellular CD3 expression [14]. For additional details on the characteristics of the ZKO cell line, see Figs. S4 and S5.

### CD247 Variants Display Contrasting Phenotypic and Functional TCR Reconstitution Levels in ZKO cells

At first, we examined TCR expression following ZKO transfection with EGFP-tagged constructions. We discovered that transfection of hCD247-WT resulted in TCR levels that were comparable to control values. Additionally, the increase in surface CD3 expression was directly proportional to the amount of EGFP expressed by the cell (Fig. S6).

Unfortunately, we were unable to assess the functional recovery of these cells due to damage caused by transfection. To overcome this issue, we cloned all CD247 variants into the pHRSIN-C56W-UbEM plasmid and utilized a lentiviral transduction protocol to carry out these assays.

**Fig. 4:**
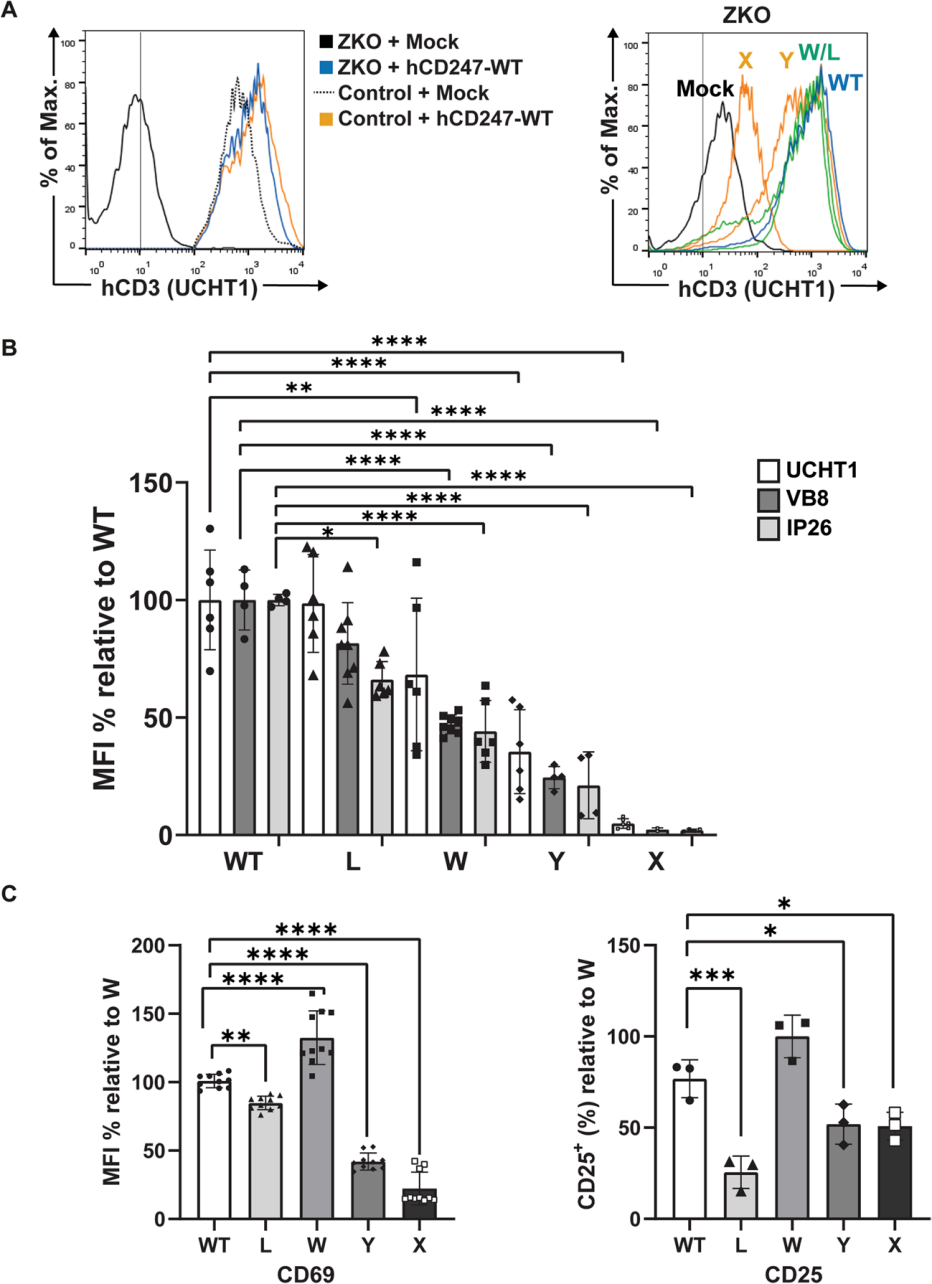
Phenotypical and functional TCR reconstitution analysis by the CD247 variants expressed in the ZKO cell line. (**A**) Representative flow cytometry analysis of surface TCR complex reconstitution in Jurkat WT (control) or ZKO cell lines transduced with the pHRSIN-C56W-UbEM plasmid carrying either nothing (Mock) or hCD247-WT (left) or the different CD247 variants (right) using anti-CD3ε (UCHT1) mAb. Transduced cells were pre-gated as GFP +. The name of each variant is indicated above its corresponding histogram (right). The vertical line represents the isotype control. (**B**) Quantification of hCD3 expression from A (mean MFI values, *n* = 6). (**C**) Quantification of surface CD69 induction (% MFI) and percentage of CD25^+^ cell induction (*n* = 3) after stimulation with SEE-loaded Raji cell conjugates. Transduced cells were gated as CD19^-^, GFP^+^. In (**B**) and (**C**), values are relative to the highest for each analyzed parameter and were normalized as (test—mock)/(highest variant—mock). In all cases, WT corresponds to hCD247-WT, X to Q70X, Y to Q70Y, W to Q70W and L to Q70L. Statistical significance was calculated using a one-way ANOVA test, *p-value < 0.05, **p-value < 0.01, ***p-value < 0.001, ****p-value < 0.0001

When hCD247-WT was introduced into the ZKO cell line through lentiviral transduction, surface TCR expression, measured with UCHT1 mAb, was completely restored (100%, compared with the Jurkat WT cell line). However, the other CD247 variants showed varying abilities to recover TCR expression, as seen in previous experiments (Fig. 4A). The quantification of these results showed that the somatic variants Q70L and Q70W had the highest reconstitution levels, with Q70L recovering 100% and Q70W recovering 70% of TCR expression when compared to hCD247-WT. On the other hand, the somatic reversion Q70Y and the germline Q70X change only recovered 37% and 7% of surface TCR levels, respectively (Fig. 4B). These results are comparable to those obtained from transfection experiments and confirmed that CD247 somatic reversions have different abilities to allow a correct TCR assembly and expression at the plasma membrane.

In addition to UCHT1, the TCR complex surface expression in the transduced ZKO cell lines was also measured with IP26 and VB8 monoclonal antibodies, which recognize the alpha–beta chains or the β-chain variable region (Vβ8) of the TCR, respectively. In general, the results obtained with IP26 and VB8 were similar to those obtained with UCHT1, as indicated by the following ranking: WT > Q70L > Q70W > Q70Y > Q70X. However, in all the variants, we observed slightly lower IP26 values than those obtained with VB8 (Fig. 4B).

Besides evaluating phenotypic TCR reconstitution, we assessed the functional reconstitution of transduced ZKO cells by observing the upregulation of T-cell activation markers upon stimulation with SEE-loaded Raji cell conjugates.

Concerning early/intermediate T-cell activation markers, specifically CD69 upregulation, our results showed that transduction of the ZKO cell line with hCD247-WT restored TCR-dependent induction of this marker after TCR engagement. Surprisingly, Q70W variant performed better than hCD247-WT in inducing CD69; whereas Q70L, which fully recovered TCR expression, was not as efficient in upregulating CD69. Q70Y, only partially induced CD69 expression, while Q70X completely blocked TCR-dependent CD69 induction (Fig. 4C). The performance of the different variants to upregulate CD69 is abbreviated as follows: Q70W > WT > Q70L > Q70Y > Q70X.

Similarly, for CD25 induction, transduction with hCD247-WT recovered the ability of the ZKO cell line to express this activation marker. Once again, the Q70W reversion resulted in better CD25 upregulation compared to Q70L. Interestingly, Q70X induced CD25 at similar levels to Q70Y (Fig. 4C). In summary, the order of CD25 induction efficiency is Q70W = WT > Q70L > Q70Y = Q70X.

Regarding the early activation event, p-ZAP70, we observed a significant increase only in hCD247-WT transduced ZKO cells. However, we also noticed a slight upregulation in Q70L, Q70Y and Q70W, while Q70X was unable to induce it (Fig. S7).

Based on our findings, we determined that the TCR phenotypical reconstitution ability of the studied CD247 variants differs from their functional reconstitution ability. Our data also showed that the CD247 somatic reversions had a poorer functional performance at short times (p-ZAP-70) but improved over time (CD69 and CD25).

To explain our results, we used AlphaFold to generate *in silico* 3D models of CD247. While the transmembrane region showed reliable predictions, the cytoplasmic region, including Q70, was intrinsically disordered, preventing accurate structural modeling (data not shown). To assess whether the Q70Y variant affects CD247-ZAP70 and CD247-LCK interactions, we performed additional *in silico* analyses. Our results showed that both Q70 and Y70 are positioned outside the ZAP70 interaction interface, suggesting no impact on CD247-ZAP70 binding (data not shown). For the CD247-LCK interactions, accurate modeling of ITAM1 and ITAM2 was not possible, but we successfully modeled ITAM3, where G139 is the position equivalent to Q70 in ITAM3, relative to the first tyrosine of ITAM3 (Y141). Introducing the G139Q maintained Y141 within the LCK active site, whereas the G139Y variant repositioned Y141 outside (Fig. S8). Although these results support the notion that the Y70 variant reduces CD247 phosphorylation by LCK, a similar result was observed with the CD247 L70 variant (data not shown). These findings suggest that Q70 plays a critical role in stabilizing or enhancing CD247-LCK interactions.

## Discussion

Somatic reversion, a phenomenon observed in various primary immunodeficiencies, significantly modifies the clinical outcomes of these pathologies [4]. It can be positive, neutral or negative, depending on the gene. Interestingly, despite the detection of immune cell subsets expressing functional proteins and exhibiting restored functionality, somatic reversion improved clinical outcomes only in certain conditions [4, 18]. For instance, it has a positive clinical impact on ADA deficiency, Wiskott-Aldrich syndrome (WAS), Fanconi anemia, and in variants affecting DOCK8, ITGB2*,* or CXCR4 [18]. However, it negatively impacts patients with somatic variants in CARD11, RAG1, as well as in a reported case of IL2RG reversion in tissue infiltrating T-cells, all associated with Omenn syndrome [4, 18]. Additionally, one patient with a reversion in NEMO, developed refractory inflammatory colitis, as his revertant T cells activated NF-kB in response to growth signals and had a growth advantage over cells carrying the germline change [19]*.* In other cases, somatic reversion can be neutral, as seen in CD247 or Ligase IV [20] deficiencies, where WT reversions were unable to modify the clinical or immunological phenotype. It has been hypothesized that somatic revertant variants are common in proliferative tissues, like the hematopoietic system, but are limited by the need for functional protein restoration [21, 22].

In the case of CD247 deficiency, only five patients with homozygous germline variants have been reported. Among these, four exhibited a small population of revertant T cells (Table S1), but this did not correlate with improved clinical outcomes as all experienced life-threatening infections, with only one surviving post-hematopoietic stem-cell transplantation (HSCT).

Since no functional studies have explored the potential effect of CD247 somatic reversions, one main objective of our study was to understand, at the molecular level, the impact of different variants on TCR surface expression and CD247-dependent T-cell functions. To this end, we chose the main CD247 somatic reversions (WT, p.Q70W, p.Q70L, and p.Q70Y) in comparison to the germline change (p.M1T, and p.Q70X) reported by Marin [13] and Rieux-Laucat [17], respectively. We found varying degrees of surface TCR expression restoration among the revertants (WT > Q70L > Q70W > Q70Y > > Q70X), which was consistent across different staining antibodies. As expected, the p.Q70X germline variant showed no restoration (Fig. 4A, B and Table S2). This suggests that the revertants can partially or fully restore TCR expression, whereas the germline variant is incapable of doing so. These differences might indicate that the specific amino acid changes in each revertant differentially affect the efficiency of TCR complex assembly and surface expression.

Notably, by testing murine and human cell lines side-by-side, we learned that CD247-deficient mouse T cells MA5.8 cannot be used to model human CD247 deficiencies, since Q70X, which is expectedly unable to restore TCR expression in human CD247-deficient T cells (PM1T or ZKO), does so in MA5.8 (Fig. 2 and Table S2). Therefore, previous reports using such murine cell lines should be reinterpreted in light of our findings [5, 23]. A potential limitation of this conclusion is that they are drawn from a truncated protein (Q70X) that is translated upon transfection and transduction, respectively, from a cDNA, and thus may not exist in primary patient’s T cells due to nonsense-mediated RNA decay (NMD), which is prevented when using cDNA. Rieux-Laucat et al., reported in the discussion that CD247 Q70X was detected in small amounts in the cytoplasm, but not on the membrane in primary patient’s T cells [17]. Although no data were included to support that contention, impaired Q70X protein expression could be partly due to NMD. We identified a CD247 Y152X nonsense variant [14, and unpublished results] in primary patient’s T cells, which was seemingly unaffected by NMD, likely due to its location in the last exon of the gene. In contrast, the reported c.301C > T (p.Gln101X) variant, which is closer to the Q70 codon, results in a premature translation-termination codon (PTC) predicted to cause a truncation of the encoded protein or absence of the protein due to NMD (Variation ID: 419227). Together, these findings confirm the impact of PTC location in NMD and suggest that the Q70X variant could be an NMD candidate. Genome-edited cell lines with Q70X are in progress to address this and other questions in more detail in the future.

Interestingly, analysis of the impact of revertant variants on TCR function, by measuring surface expression of CD69 and CD25 after stimulation with SEE-loaded Raji cells, revealed patterns of CD69 upregulation (Q70W > WT > Q70L > Q70Y > > Q70X) (Fig. 4C, left) and CD25 + cells (Q70W > WT > Q70Y = Q70X > Q70L) (Fig. 4C, right) which did not correlate with surface TCR expression. The p.Q70W variant showed the highest functional restoration, while the p.Q70L variant, despite good TCR expression recovery, was less effective in inducing CD25 expression upon TCR engagement. These results indicate that the p.Q70W variant, although not the best at restoring TCR expression, is the most effective in triggering T cell activation, as shown by higher TCR-mediated CD69 upregulation. We hypothesize that Q70W outperforms wild-type (WT) and Q70L in CD69 and CD25 upregulation due to its aromatic ring, which may facilitate stronger interactions with signaling partners, enhancing ITAM phosphorylation and activation. In contrast, Q70L hydrophobic nature likely hinders essential hydrogen bonding, impairing ITAM accessibility and phosphorylation. This lack of interactions may impair early/intermediate activation events, including CD69 upregulation and sustained CD25 induction. This further suggests that certain CD247 reversions may enhance signaling pathways downstream of the TCR, leading to more robust T-cell activation.

*In silico* predictors PolyPhen, SIFT or Saphetor classified Q70X as strongly pathogenic, Q70W and Q70Y as probably damaging, while Q70L is considered benign. Therefore, the expected clinical hierarchy based on these predictors would be (WT = Q70L > Q70W = Q70Y > > Q70X). Our phenotypic results generally confirm but also refine such predictions (WT > Q70L > Q70W > Q70Y > > Q70X). The functional results add further layers of complexity, as expression is required for function: Q70W > WT > Q70L > Q70Y > > Q70X (by CD69 upregulation), Q70W > WT > Q70Y = Q70X > Q70L (by CD25^+^ cells) and WT > > Q70L = Q70W = Q70Y > > Q70X (by Zap70 phosphorylation, as reported by Rieux-Laucat [17]). We thus believe that our cellular model to interrogate variants is more informative than purely *in silico* predictors.

In addition, the discrepancy between the TCR expression levels and the degree of TCR activation, suggests that Q70 substitutions impact CD247 function beyond surface expression. Q70, a polar residue, likely stabilizes CD247 via hydrogen bonding. Replacing it with hydrophobic (leucine and tryptophan) or amphipathic (tyrosine) residues may alter conformation and signaling. Q70L supports TCR expression but weakens activation, likely due to the loss of hydrogen bonding crucial for ITAM phosphorylation. Q70W disrupts surface expression but enhances signaling, possibly by facilitating stronger ITAM interactions through its aromatic ring. Q70Y impairs both expression and early activation, likely because its bulky aromatic ring and polar hydroxyl group disrupt the structural integrity or proper folding of CD247. However, its hydroxyl group may allow limited interactions that support moderate CD25 expression, indicating partial preservation of sustained signaling pathways.

Given the crucial role of CD247 in TCR selection and tolerance, the clinical implications of the results obtained with the Q70W and Q70L variants are significant. The Q70W heightened induction of CD69 and CD25 could potentially lead to excessive T-cell activation, disrupting the immune balance and increasing the risk of autoimmune diseases and chronic inflammation. Conversely, the Q70L variant, with its impaired T-cell activation, may result in immunodeficiency, increasing susceptibility to infections and related complications.

In addition, none of the CD247 revertants or the germline change induced ZAP-70 phosphorylation after TCR stimulation with an anti-CD3 mAb (Fig. S7 and Table S2). This can be attributed to the fact that ZAP-70 phosphorylation is an immediate (very early) event during T cell activation, whilst CD69 and CD25 upregulation are early events, relatively distal from TCR signaling initiation (which peak at 24 and 48 h post-stimulation, respectively); thus, allowing the cell to respond to cumulative TCR signaling from CD247 somatic variants, or, alternatively, from CD3 receptor subunits [24].

Overall, these findings highlight that restoring TCR expression does not always lead to functional recovery, and each revertant shows a unique restoration profile, underlining the importance of evaluating multiple functional markers to fully understand the impact of somatic reversions. However, we are fully aware that for missense variants, overexpression from strong promoters can compensate for the functional defects of the protein, so it would be advisable for this kind of experiments, to generate cell lines that express the variants of interest from the endogenous gene and not generating knock-out cell lines that are transduced with a cDNA that is overexpressed.

Concerning the discordant effects on expression and function of the studied variants, Kaiser et al*.* [21] reported a CD247-deficient patient (Table S1) with a frameshift change in the CD247 leader peptide, exhibiting more than 30 non-WT somatic variants that could, to varying degrees, restore surface TCR expression. Some of them persisted for months and some others did not, irrespectively of surface TCR expression levels, suggesting the existence of discordant effects on expression and function (measured as T cell survival *in vivo*), although such effects were not operating in TCR assembly interactions, but rather in leader peptide functions. In other genes, some examples have been reported, too. Reconstitution assays in vitro with pre-TCRα variants found in patients showed that some restored surface pre-TCRα expression partially but were fully impaired functionally [25]. Similarly, expression of a NEMO variant protein was not markedly reduced by flow cytometry, but its activity was defective, as confirmed by an NF-κB luciferase reporter assay [19].

In conclusion, our results indicate that somatic mosaicism in CD247 is a common, random event in T cells. However, its capacity to restore TCR expression does not match TCR function. Additionally, none of the tested revertants, including WT, improved the patients’ survival, likely because these events took place too late in T cell development to have a clinical impact, as suggested by Kaiser [21] and Attardi [22]. These findings may be relevant to understand the role of CD247 in TCR structure and function during human T cell development *in vivo* and its impact on human immunodeficiencies.

## Supplementary Information

Below is the link to the electronic supplementary material.

Supplementary file1 (PDF 1288 KB)

## Data Availability

No datasets were generated or analysed during the current study.
