# Supplementary material for: Discordant Restoration of TCR Expression and Function by CD247 Somatic Reversions
Source: J Clin Immunol. 2025 Jul 25;45(1):116. doi: 10.1007/s10875-025-01908-9 (PMC12296992; doi:10.1007/s10875-025-01908-9)
Supplement: Supplementary file 1 — Supplementary file1 (PDF 1288 KB) [file 10875_2025_1908_MOESM1_ESM.pdf]

Supplementary Information

Journal of Clinical Immunology

Discordant restoration of TCR expression and function by CD247 somatic reversions

Alejandro C. Briones[1], Ana V Marin[1], Rebeca Chaparro-García[1], Marta López-Nevado[1], David Abia[2], Ivan Estevez-Benito[1], Daniel Chacón-Arguedas[1], Edgar Fernández-Malavé[1], Paula P. Cardenas[1*], José R. Regueiro[1*].

1- Department of Immunology, Ophthalmology and ENT, Complutense University School of Medicine and 12 de Octubre Health Research Institute (imas12), Madrid, Spain

2- Bioinformatics Unit, Centro de Biología Molecular Severo Ochoa (CSIC-UAM), Campus of the Universidad Autónoma de Madrid, Madrid, Spain

* Contributed equally

*****Corresponding authors: Jose R. Regueiro, regueiro@ucm.es; Paula P. Cardenas, paulcard@ucm.es (Department of. Immunology, Ophthalmology and ENT; School of Medicine; Complutense University; 28040 Madrid, SPAIN

Methods

Generation of a CD247-deficient human cell line, PM1T

Allogeneic in vitro expanded CD3high (CD247 haploinsuficient) and CD3low (CD247 deficient) cells from a CD247-deficient patient **[1]** were used for in vitro HTLV-1 generation as described **[2]**. This patient showed marked T- and B- cell lymphopenia and reduced surface TCR expression in αβ and γδ T cells caused by the homozygous c.2T>C, p.M1T change, which prevented protein translation. Although most T cells had very low TCR levels (CD3low), a small population (0,2%) had surface TCR levels similar to control ones (CD3high). To isolate the CD3low population, PBMC from the patient were allogeneic cultured and sorted into CD3low and CD3high. Western blot with anti-CD247 antibody confirmed that the CD3low subpopulation did not express CD247 protein (data not shown). This population was immortalized after infection with Human T-cell leukemia virus type 1 (HTLV-1), as reported previously **[3]**. Once the CD247-deficient human cell line was established, it was named PM1T, as for the change it harbors. Genomic DNA sequencing confirmed that the patient's original change remained in the PM1T cell line (data not shown).

Western Blotting

Cells were lysed in RIPA buffer, as published elsewhere, and 40 µg protein per sample, determined with *DC* Protein Assay (Bio-Rad, Hercules, CA, USA), were loaded into a 12% SDS-PAGE gel.
Following electrophoresis, the gel was blotted onto a PVDF membrane and developed with the following anti-CD247 antibodies: mouse mAb 6B10.2 (BioLegend); rabbit polyclonal 448, specific for residues 109-132 of human CD247 **[4]**, gifted by Balbino Alarcón. Anti-α-tubulin (clone B-5-1-2) from Sigma-Aldrich (St. Louis, MO) was used as a loading control.

Results

Characterization of human and mouse CD247-deficient cell lines

Since no CD247-deficient human cell line was available, the well-characterized murine T-cell hybridomas, 2B4.11, and its CD247-deficient derivative MA5.8 **[5]**, widely used for CD247 reconstitution assays **[6,7]**, were used as controls for subsequent experiments.
To characterize our cell lines, we examined the expression of CD247 in human (PM1T and control) and murine (2B4.11 and MA5.8) cell lines. To this end, we performed a Western blot revealed with 448 antibody, which recognizes both human and mouse CD247 **[8]**. We found that CD247 expression was not detected in either PM1T or MA5.8 lysates, confirming that its synthesis was abrogated in both cell lines. However, CD247 was present in their respective controls **(Fig. S2A)**.
Next, the effect of CD247 deficiency on membrane TCR expression was analyzed by flow cytometry with anti-CD3 antibodies. We found that PM1T maintained the defective TCR surface levels of the patient's $CD3^{low}$ population, with only 4% CD3 expression compared to its HTLV-1 immortalized control. A similar value, 12%, was obtained after comparing CD3 expression in MA5.8 to its parental line 2B4 **(Fig. S2B)**.
In addition, the PM1T cell line displayed abnormal T-cell function as it could not induce CD69 upregulation in a TCR-dependent response following anti-CD3 antibody stimulation. However, TCR-independent responses induced by PMA-Ionomycin resulted in increased CD69 levels **(Fig. S2C)**.

Characterization of the CD247-deficient Jurkat cell line (ZKO)

In addition to the reduced CD3 extracellular expression, the intracellular levels of CD3ε in ZKO were 66% compared with its control cell line **(Fig. S4A)**. These data indicate that CD247 is necessary for the optimal assembly and transport of TCR complexes to the cell surface.
To assess the impact of CD247 absence on T cell activation, we examined the upregulation of CD69 in control and ZKO cells in response to TCR-dependent (OKT3) and -independent (PMA-Iono) stimuli. The results showed that the ZKO cell line only responded to TCR-independent signals, while the Jurkat WT control cell line was able to respond to both types of stimuli **(Fig. S4B and Fig. S5)**. These findings suggest that the ZKO cell line has a significant functional impairment similar to that observed in the PM1T cell line and primary T lymphocytes from the CD247-deficient patient carrying the PM1T germline change. Furthermore, these results remark the essential role of CD247 in transducing signals after T-cell activation.

Tables

Table S1. Clinical and immunological features of patients with reported CD247 deficiency					
**Case No.**	**1**	**2**	**3**	**4**	**5**
**Born in**	Turkey	Micronesia	Caribbean	Not reported	India
**Sex**	Female	Female	Male	Female	Female
**Diagnosis (m)**	2	11	4	22	24
**Consanguinity**	Yes	No	Not reported	Yes	Yes
**Autoimmunity**	No	Yes (ITP)	No	Yes (JRA, ES)	Yes (ES)
**Germline mut.**	c.2T>C	c.411insC	c.208C>T	c.43_44delCA	c.62C>T
**Predicted prot.**	p.M1T	p.D138fsX272	p.Q70X	p.Q15VfsX72	p.A21V
**Lymphocytes/uL, 2**	**3000**	**141**	**1200**	**1200**	5160
**CD3+ cells/uL, 2**	**1320**	**90**	**252**	**300**	**103**
**HSCT at (m or y)**	19 m	12 m, 16m…	30 m	10 y	No
**Alive**	No	No*	Yes	No	No
**Cause of death**	Pneumonia	Unknown	—	—	Pneumonia
**Revertant cells?**	Yes	Yes	Yes	Yes	Not reported
**$CD3^{High}$ cells / uL**	2	1	10	22	Not reported
**% surface TCR, 4**	50	40	Not reported	44	Not reported
**CD4/8 phenotype**	CD4 > CD8	CD4 > CD8	CD4	CD4 > CD8	Not reported
**Functional?, 3**	Yes (=carriers)	Not reported	No	Not reported	Not reported
**Revertant mut.**	2 (=wild type)	Not reported	3 (non-WT)	> 40 (non-WT)	Not reported
**Reference**	Marin 2017 [1]	Roberts 2007 [6]	Rieux 2006 [9]	Kaiser 2021 [10]	Setia 2021 [11]
Notes					
1- ITP= Idiopathic Thrombocytopenic Purpura, JRA=Juvenile rheumatoid arthritis,					
ES= Evan Syndrome: autoimmune hemolytic anemia and thrombocytopenia					
2- **Bold means out of range** (normal age-matched ranges from Schatorjé[12])					
3- Early phosphorylation					
4- Relative to healthy donors (estimated data). In case No.3 % intracellular staining was 60%					
* Personal communication					

Table S1 Clinical and immunological features of patients with reported CD247 deficiency.

STUDIED PARAMETER	WT	L	W	Y	X	MOCK	UNTR	DEFICIENT CELL LINES	TECHNIQUES	mAb	SPECIES
SURFACE TCR EXPRESSION	100	102	100	35	94	0	12	MA5.8	NUCLEOF	2C11	MOUSE
	100	98	100	61	0	0	4	PM1T	NUCLEOF	UCHT1	HUMAN
	100	132	100	52	-2	0/11	4	PM1T	TRANSD	UCHT1	HUMAN
	100	99	68	35	5	0	5	ZKO	TRANSD	UCHT1	HUMAN
ZAP-70 PHOSPHORYLATION	100	28	27	25	5,5	0/1,0		ZKO	TRANSD	p-ZAP70	HUMAN
CD69 UPREGULATION	75	60	100	25	8	0/12,5		ZKO	TRANSD	CD69	HUMAN
% CD25+ CELLS	100	33	130	68	66	0/15,3		ZKO	TRANSD	CD25	HUMAN
CD25 UPREGULATION	100	-29	88	24	21	0/65		ZKO	TRANSD	CD25	HUMAN
**STUDIED PARAMETER**	**WT**	**L**	**W**	**Y**	**X**	**MOCK**	**UNTR**	**WT CELL LINES**	**TECHNIQUES**	**mAb**	**SPECIES**
SURFACE TCR EXPRESSION	Not performed				100	100	ND	2B4	NUCLEOF	2C11	MOUSE
	178	175	147	57	82	100	ND	HTLV1	TRANSD	UCHT1	HUMAN
	Not performed				10	100	ND	JURKAT	NUCLEOF	UCHT1	HUMAN

Table S2 Comparison of the results obtained for the analyzed parameters (TCR expression, p-ZAP70, CD69 and CD25 upregulation, and percentage of CD25 positive cells [%CD25+]) across the studied CD247 variants (WT, Q70L/W/Y/X). This analysis was performed on cell lines of different origins (human or mouse; CD247-deficient or CD247-sufficient [black or red letters, respectively]) and using distinct gene transfer techniques (nucleofection or transduction). UNTR: Not nucleofected or transduced. ND: Not determined.

Figures

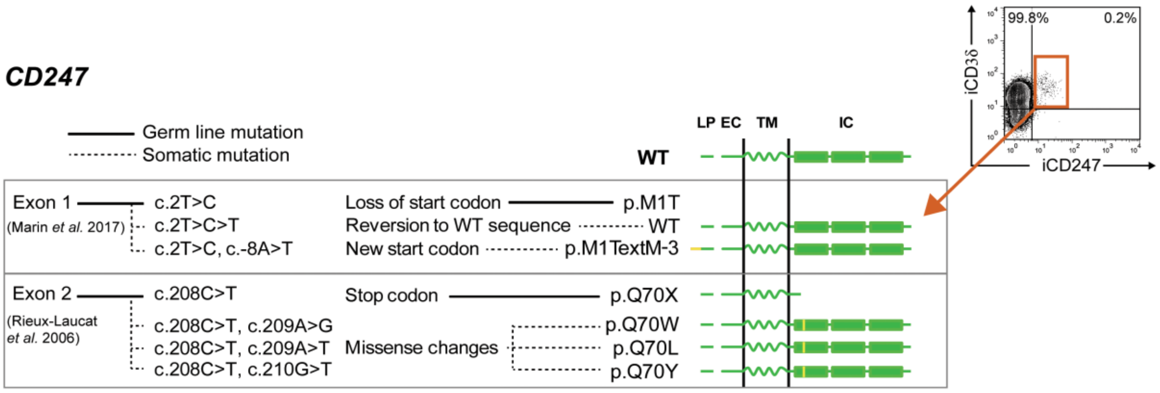

Figure S1 Schematic representation of two germline CD247 changes, where a subpopulation of revertant T cells due to the indicated somatic variants was reported. The inset shows an example of intracellular staining for CD3**δ** and CD247 of primary T cells. The red square highlights revertant cells, and the arrow indicates the revertant changes found in that case (adapted from Marin **[1]**).

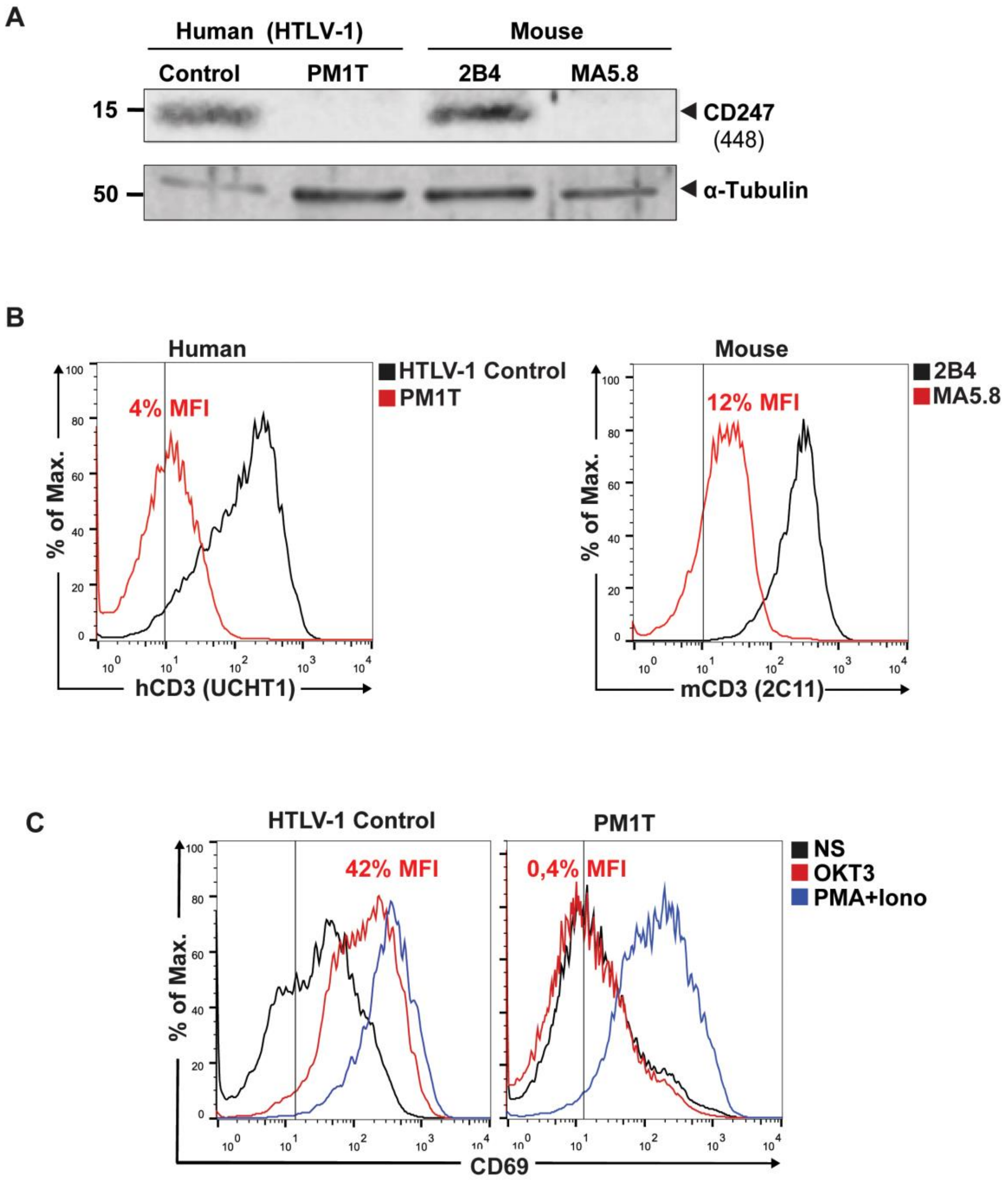

Figure S2 Characterization of human and mouse CD247-deficient cell lines. **(A)** Western blot showing CD247 expression levels in human HTLV-1 immortalized (Control and PM1T) and murine (2B4 and MA5.8) cell lines revealed with the 448 antibody. α-Tubulin was used as a loading control. **(B)** CD3ε surface levels in CD247-sufficient (Control and 2B4) and –deficient (PM1T and MA5.8) cell lines measured by flow cytometry using mAbs against CD3ε (clones UCHT1 or 2C11 for human or mouse cell lines, respectively). The upper value indicates the percentage of surface TCR expression in the deficient cell line with respect to its corresponding control cell line. **(C)** Flow cytometry analysis of CD69 upregulation in human HTLV-1 immortalized PM1T and control cell lines after TCR-dependent (anti-CD3 antibody, OKT3) or TCR -independent stimulation (PMA-Iono). NS: Non-stimulated. For both **(B)** and **(C)**, the vertical line represents the isotype control. The histograms correspond to a single representative experiment (from 4 independent experiments).

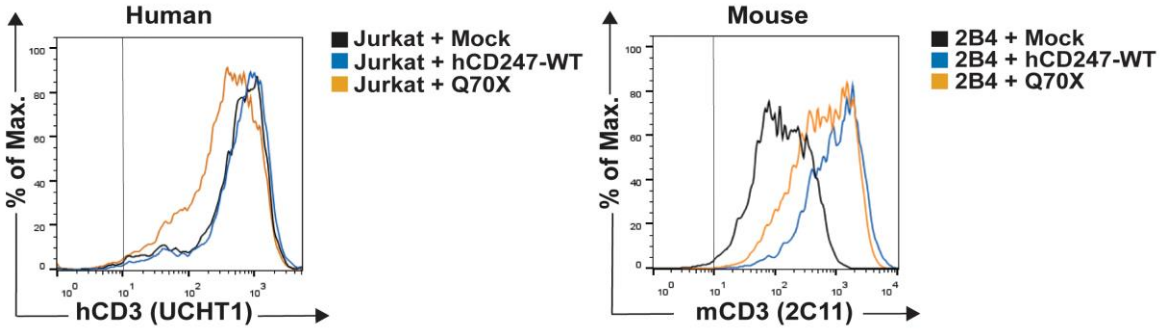

Figure S3 Impact of Q70X change on surface TCR expression in human or mouse control T cells. Human (Jurkat) or mouse (2B4) control T cell lines were transfected with hCD247-WT or Q70X variant. Surface CD3 expression was analyzed with mAbs against hCD3 (UCHT1) or mCD3 (2C11). The vertical line corresponds to the isotype control. The graphics represent the results of one experiment.

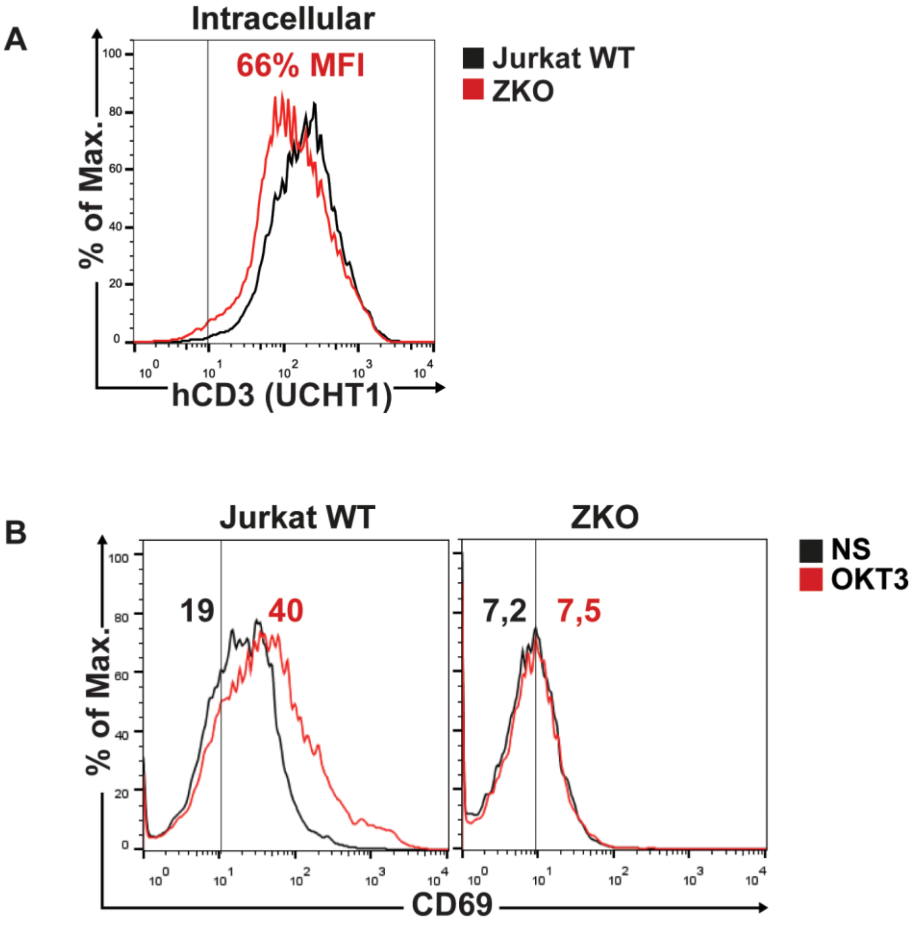

Figure S4 Biochemical and functional characterization of the CD247-deficient Jurkat cell line (ZKO). **(A)** Intracellular flow cytometry staining of Jurkat WT (control) and ZKO cell lines with CD3ε mAb (UCHT1). The upper value indicates the MFI % compared to the control cell line. **(B)** Flow cytometry analysis of CD69 upregulation in WT and ZKO Jurkat cell lines after TCR-dependent stimulation (anti-CD3 antibody, clone OKT3). The numbers indicate MFI values for each condition. NS: Non-stimulated. In all flow cytometry experiments, the vertical line represents the isotype control. Only one representative experiment is depicted (from 3 independent experiments).

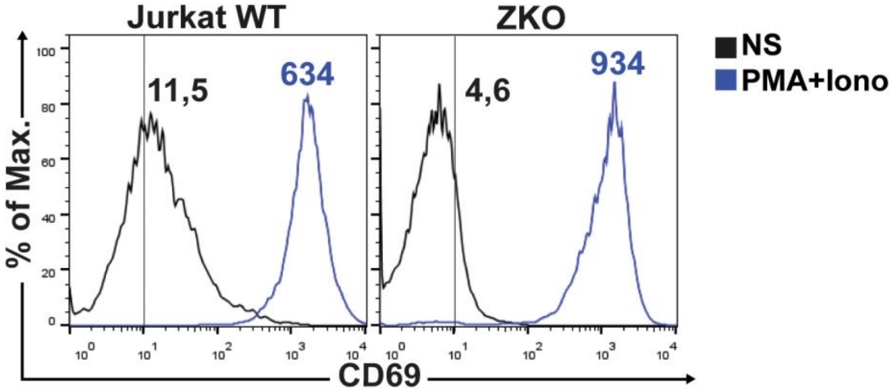

Figure S5 Flow cytometry analysis of CD69 upregulation in WT and ZKO Jurkat cell lines after TCR-independent stimulation (PMA+Iono). The numbers indicate the MFI values. Only one representative experiment is depicted (from 2 independent experiments).

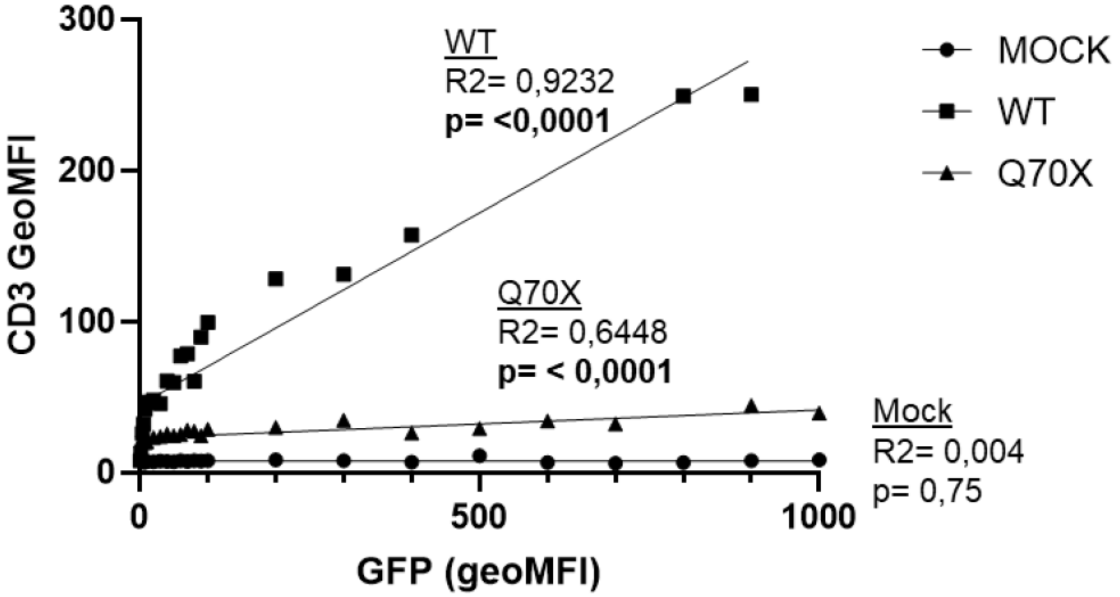

Figure S6 Surface TCR/CD3 reconstitution using CD247 constructs in Jurkat ZKO cells. The cells were transfected by nucleofection with empty pEGFP-N1 (MOCK), hCD247-WT or Q70X, EGFP fusion variants and surface TCR expression levels were quantified by FACS along sequential GFP levels intervals. Simple linear regression for each variant was calculated.

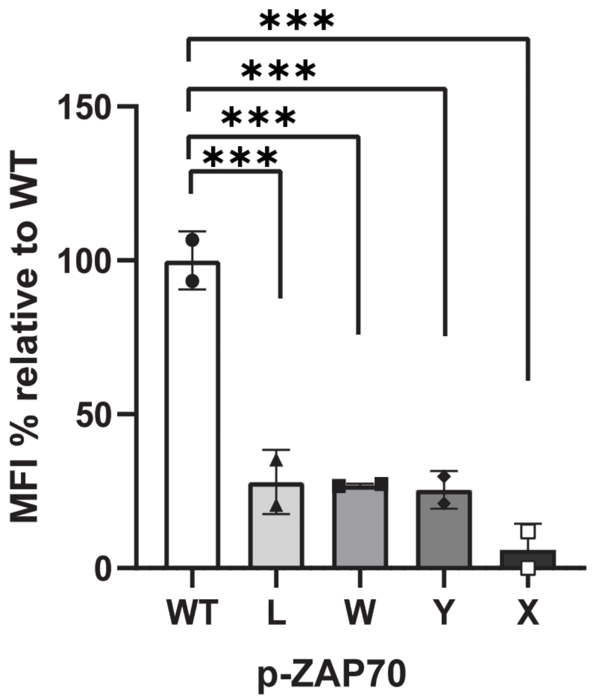

Figure S7 Induction of p-ZAP70 in ZKO cell lines transduced with the studied CD247 variants. Intracellular flow cytometry staining of transduced ZKO cell lines with anti-ZAP70/Syk (Tyr319, Tyr352) mAb. Quantification of p-ZAP-70 expression normalized with respect to transduced hCD247-WT (n=2). Statistical significance was calculated using a one-way ANOVA test. ***p-value <0.001.

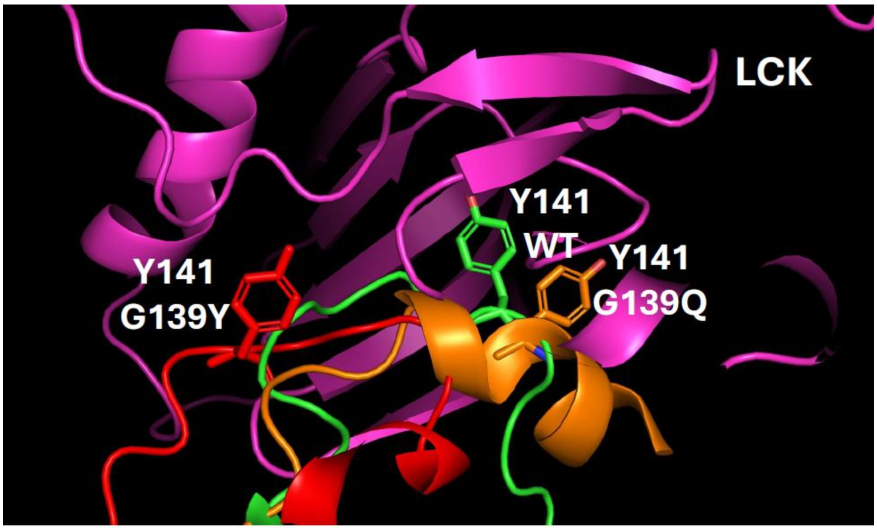

Figure S8 In silico 3D model of the CD247 ITAM3-LCK interaction predicted by AlphaFold. The image shows the LCK active site (fuchsia) and residue Y141 in CD247-WT (green), as well as in CD247 with variants G139Q (orange) and G139Y (red).

References

1. Marin AV, Jiménez-Reinoso A, Briones AC, Muñoz-Ruiz M, Aydogmus C, Pasick LJ, et al. Primary T-cell immunodeficiency with functional revertant somatic mosaicism in CD247. J Allergy Clin Immunol 2017;139:347-349.e8. https://doi.org/10.1016/j.jaci.2016.06.020
2. Marin AV, Jiménez-Reinoso A, Mazariegos MS, Román-Ortiz E, Regueiro JR. T-cell receptor signaling in Schimke immuno-osseous dysplasia is SMARCAL1-independent. Front Immunol. 2022;13:979722.https://doi.org/10.3389/fimmu.2022.979722
3. Nutman TB. Generation of HTLV-I-transformed T cell lines. Curr Protoc Immunol. 2002;Chapter 7:Unit 7.20. https://doi.org/10.1002/0471142735.im0720s47
4. Sahuquillo AG, Roumier A, Teixeiro E, Bragado R, Alarcón B. T Cell Receptor (TCR) Engagement in Apoptosis-defective, but Interleukin 2 (IL-2)–producing, T Cells Results in Impaired ZAP70/CD3-ζ Association. J Exp Med 1998;187:1179–92. https://doi.org/10.1084/jem.187.8.1179
5. Sussman JJ, Bonifacino JS, Lippincott-Schwartz J, Weissman AM, Saito T, Klausner RD, et al. Failure to synthesize the T cell CD3-zeta chain: structure and function of a partial T cell receptor complex. Cell 1988;52:85–95. https://doi.org/10.1016/0092-8674(88)90533-8
6. Roberts JL, Lauritsen JPH, Cooney M, Parrott RE, Sajaroff EO, Win CM, et al. T-B+NK+ severe combined immunodeficiency caused by complete deficiency of the CD3zeta subunit of the T-cell antigen receptor complex. Blood 2007;109:3198–206. https://doi.org/10.1182/blood-2006-08-043166
7. Blázquez-Moreno A, Pérez-Portilla A, Agúndez-Llaca M, Dukovska D, Valés-Gómez M, Aydogmus C, et al. Analysis of the recovery of CD247 expression in a PID patient: insights into the spontaneous repair of defective genes. Blood 2017;130:1205–8. https://doi.org/10.1182/blood-2017-01-762864
8. Fernández-Malavé E, Wang N, Pulgar M, Schamel WWA, Alarcón B, Terhorst C. Overlapping functions of human CD3δ and mouse CD3γ in αβ T-cell development revealed in a humanized CD3γ-deficient mouse. Blood 2006;108:3420–7. https://doi.org/10.1182/blood-2006-03-010850
9. Rieux-Laucat F, Hivroz C, Lim A, Mateo V, Pellier I, Selz F, et al. Inherited and somatic CD3zeta mutations in a patient with T-cell deficiency. N Engl J Med 2006;354:1913–21. https://doi.org/10.1056/nejmoa053750
10. Kaiser FMP, Reisli I, Pico-Knijnenburg I, Langerak AW, Kavelaars FG, Artac H, et al. Protein functionality as a potential bottleneck for somatic revertant variants. J Allergy Clin Immunol 2021;147:391-393.e8. https://doi.org/10.1016/j.jaci.2020.04.045
11. Setia P, Bargir UA, Aluri J, Sampagar A, Pandit A, Kumar V, et al. Novel CD3Z and CD3E Deficiency in Two Unrelated Females. J Clin Immunol 2021;41:1116–8. https://doi.org/10.1007/s10875-021-01010-w
12. Schatorjé EJH, Gemen EFA, Driessen GJA, Leuvenink J, Van Hout RWNM, De Vries E. Paediatric Reference Values for the Peripheral T cell Compartment. Scand J Immunol 2012;75:436–44. https://doi.org/10.1111/j.1365-3083.2012.02671.x
